# Superior Cardiorenal Protection With Tirzepatide Over Dulaglutide in Heart Failure With Preserved Ejection Fraction: A Large‐Scale Propensity Score–Matched Real‐World Analysis

**DOI:** 10.1002/clc.70441

**Published:** 2026-08-10

**Authors:** Ibrahim Khalil, Md. Imran Hossain, Mst. Mahmuda Akter, Ferdous Wahid, Aizaz Anwar Khalid, Sunjida Amin Promi, Md. Abu Sayed, Mohd Turzo Rahman, Pallab Sarker

**Affiliations:** ^1^ Dhaka Medical College and Hospital Dhaka Bangladesh; ^2^ Manikganj Medical College and Hospital Manikganj Bangladesh; ^3^ Dinajpur Medical College and Hospital Dinajpur Rangpur Bangladesh; ^4^ Peshawar Medical College Peshawar Pakistan; ^5^ Chattogram Medical College and Hospital Chattogram Bangladesh; ^6^ Department of Internal Medicine Flushing Hospital Medical Center New York New York USA; ^7^ Department of Internal Medicine Reading Hospital West Reading Pennsylvania USA

**Keywords:** dulaglutide, GLP‐1/GIP receptor agonist, heart failure with preserved ejection fraction, propensity score matching, tirzepatide

## Abstract

**Background:**

No direct head‐to‐head trials compare tirzepatide and dulaglutide in heart failure with preserved ejection fraction (HFpEF), though tirzepatide shows superior weight loss and potential cardiovascular benefits in recent trials like SURPASS‐CVOT.

**Methods:**

This retrospective, propensity score‐matched cohort study used de‐identified electronic health records from the TriNetX US Collaborative Network (as of December 2025). Adults ≥ 18 years with HFpEF (ICD‐10: I50.3) initiating tirzepatide (*n* = 35 174) or dulaglutide (*n* = 52 999), without prior exposure to the comparator, were identified. One‐to‐one greedy nearest‐neighbor matching (caliper 0.1) on demographics, comorbidities, medications, and laboratory/echocardiographic data produced balanced cohorts of 24 305 patients each. Kaplan–Meier and Cox proportional hazards models evaluated 1‐year and 3‐year risks of all‐cause mortality, acute heart failure events, all‐cause hospitalization, myocardial infarction, stroke, atrial fibrillation, MACE, MAKE, acute pancreatitis, and hypoglycemia.

**Results:**

Post‐matching, cohorts were well‐balanced. Tirzepatide linked to lower 1‐year risks of all‐cause mortality (HR: 0.66, 95% CI: [0.59–0.74]), all‐cause hospitalization (HR: 0.85, 95% CI: [0.82–0.88]), stroke (HR: 0.92, 95% CI: [0.87–0.97]), MACE (HR: 0.89, 95% [CI: 0.84–0.93]), MAKE (HR: 0.85, 95% CI: [0.79–0.91]), and acute pancreatitis (HR: 0.78, 95% CI: [0.63–0.96]). Benefits amplified at 3 years, especially mortality (HR: 0.60, [0.55–0.65]). Tirzepatide showed slightly higher 1‐year atrial fibrillation risk (HR: 1.04, [1.00–1.07]). No differences in myocardial infarction or hypoglycemia.

**Conclusions:**

In this large real‐world propensity‐matched analysis, tirzepatide demonstrated superior reductions in mortality, hospitalizations, and cardiorenal outcomes versus dulaglutide in HFpEF patients, with a favorable safety profile.

## Graphical abstract

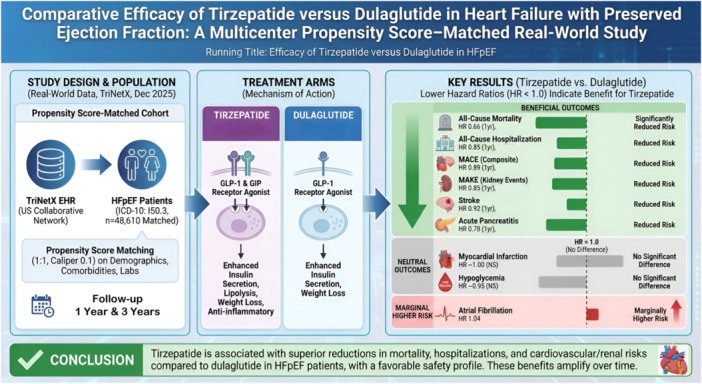

**Abbreviations**
ACEI: Angiotensin‐Converting Enzyme Inhibitor
ARB: Angiotensin Receptor Blocker
ARR: Absolute Risk Reduction
BMI: Body Mass Index
CI: Confidence Interval
COPD: Chronic Obstructive Pulmonary Disease
CPT: Current Procedural Terminology
CrI: Credible Interval
CV: Cardiovascular
CVOT: Cardiovascular Outcome Trial
eGFR: Estimated Glomerular Filtration Rate
EHR: Electronic Health Record
GDPR: General Data Protection Regulation
GIP: Glucose‐Dependent Insulinotropic Polypeptide
GLP‐1: Glucagon‐Like Peptide‐1
GLP‐1RA: Glucagon‐Like Peptide‐1 Receptor Agonist
HCO: Healthcare Organization
HCPCS: Healthcare Common Procedure Coding System
HFpEF: Heart Failure with Preserved Ejection Fraction
HFrEF: Heart Failure with Reduced Ejection Fraction
HIPAA: Health Insurance Portability and Accountability Act
HR: Hazard Ratio
ICD‐10: International Classification of Diseases, 10th Revision
ICD‐10‐CM: International Classification of Diseases, 10th Revision, Clinical Modification
KM: Kaplan‐Meier
LDL: Low‐Density Lipoprotein
LVEF: Left Ventricular Ejection Fraction
MACE: Major Adverse Cardiovascular Events
MAKE: Major Adverse Kidney Events
NNT: Number Needed to Treat
NT‐proBNP: N‐Terminal pro‐B‐Type Natriuretic Peptide
NYHA: New York Heart Association
RCT: Randomized Controlled Trial
SGLT2: Sodium‐Glucose Cotransporter‐2
SMD: Standardized Mean Difference
SNOMED: Systematized Nomenclature of Medicine
STROBE: Strengthening the Reporting of Observational Studies in Epidemiology
SUCRA: Surface Under the Cumulative Ranking Curve
T2DM: Type 2 Diabetes Mellitus

## 1 Introduction

Heart failure with preserved ejection fraction (HFpEF) now constitutes over half of all heart failure cases, representing a major unmet need in cardiovascular medicine [1, 2]. In contrast to heart failure with reduced ejection fraction (HFrEF), where multiple therapies have markedly improved outcomes, HFpEF has historically lacked disease‐modifying treatments that consistently reduce mortality or hospitalizations until the emergence of sodium‐glucose cotransporter‐2 (SGLT2) inhibitors [3]. The complex pathophysiology of HFpEF involves systemic inflammation, coronary microvascular dysfunction, myocardial fibrosis, epicardial fat accumulation, and a high prevalence of comorbidities such as obesity, type 2 diabetes mellitus (T2DM), hypertension, atrial fibrillation, and chronic kidney disease [4, 5, 6].

Glucagon‐like peptide‐1 receptor agonists (GLP‐1RAs) have recently revolutionized management in obese HFpEF. The STEP‐HFpEF trials demonstrated that once‐weekly semaglutide 2.4 mg significantly enhanced health‐related quality of life (Kansas City Cardiomyopathy Questionnaire Clinical Summary Score improvement), physical performance (6‐min walk distance), and a hierarchical composite endpoint, driven by approximately 10.7% (95% CI: 11.9 to 9.4; *p* < 0.001) weight loss in HFpEF patients with obesity, independent of diabetes [7, 8]. Building on this, the dual GLP‐1/glucose‐dependent insulinotropic polypeptide (GIP) receptor agonist tirzepatide has shown even more profound benefits, including greater reductions in heart failure hospitalizations and cardiovascular death in similar populations [9].

Dulaglutide and tirzepatide stand out as key incretin‐based therapies with robust evidence from cardiovascular outcome trials (CVOTs) in T2DM. In the REWIND trial (*n* = 9901), once‐weekly dulaglutide (a GLP‐1–albumin–Fc fusion protein with a ~ 5‐day half‐life) reduced the primary three‐point major adverse cardiovascular events (MACE) endpoint (cardiovascular death, non‐fatal myocardial infarction, non‐fatal stroke) by 12% (HR: 0.88; 95% CI: 0.79–0.99; *p* = 0.026) versus placebo, with benefits extending to patients without established cardiovascular disease [10]. Tirzepatide, a first‐in‐class dual GLP‐1/GIP agonist with a ~5‐day half‐life, has demonstrated superior glycemic control and weight loss compared to GLP‐1RAs alone in the SURPASS program [11]. However, the landmark SURPASS‐CVOT trial represents the pinnacle of evidence for tirzepatide's cardiovascular profile [12].

SURPASS‐CVOT, the most recent and comprehensive head‐to‐head CVOT in this domain, randomized 13 299 patients with T2DM and atherosclerotic cardiovascular disease to weekly tirzepatide (up to 15 mg) or dulaglutide (1.5 mg) in a double‐blind, noninferiority design [12]. This trial directly compared tirzepatide's dual agonism against dulaglutide's established GLP‐1RA efficacy, addressing uncertainties in cardiovascular outcomes beyond glycemic and weight effects. The primary composite endpoint of cardiovascular death, myocardial infarction, or stroke occurred in 12.2% of tirzepatide recipients versus 13.1% with dulaglutide (HR: 0.92; 95.3% CI: 0.83–1.01; *p* = 0.003 for noninferiority; *p* = 0.09 for superiority), confirming tirzepatide's noninferiority and suggesting a trend toward superiority [12]. Notably, tirzepatide's effects were achieved in a high‐risk cohort (mean age 64.1 years, 29% women, mean BMI 32.6, glycated hemoglobin 8.4%, diabetes duration 14.7 years), with similar adverse event profiles except for more gastrointestinal issues with tirzepatide [12]. This trial underscores tirzepatide's potential advantages, including enhanced weight loss (11.6% vs. 4.8% with dulaglutide) and dual receptor activation, which may amplify anti‐inflammatory, natriuretic, and metabolic benefits critical in HFpEF [13, 14].

Despite these advances, dulaglutide remains widely prescribed due to its proven MACE reduction and convenience [10]. Tirzepatide and dulaglutide differ fundamentally: tirzepatide's GIP co‐agonism enhances insulin secretion, suppresses appetite more potently, and may confer unique cardioprotective effects via improved lipid metabolism and endothelial function [15, 16]. Head‐to‐head trials in T2DM (e.g., SURPASS‐1, SURPASS‐2) have shown tirzepatide's superior HbA1c reduction and weight loss [11, 17]. These differences could be amplified in HFpEF, where weight reduction, inflammation attenuation, and diastolic improvement are pivotal [18].

Neither agent has been directly compared in dedicated HFpEF trials beyond SURPASS‐CVOT's broader cardiovascular focus, and no randomized study has evaluated their comparative efficacy in this syndrome [12]. With SURPASS‐CVOT providing the strongest evidence of tirzepatide's noninferiority, and potential superiority to dulaglutide in high‐risk T2DM [12], real‐world data are crucial to bridge this gap, informing guidelines and practice. Using the TriNetX US Collaborative Network (> 250 million patients), we conducted a large propensity score–matched cohort study comparing 1‐year and 3‐year risks of all‐cause mortality, acute heart failure events, all‐cause hospitalization, myocardial infarction, stroke, atrial fibrillation, major adverse cardiovascular events (MACE), major adverse kidney events (MAKE), acute pancreatitis, and hypoglycemia in patients with heart failure with preserved ejection fraction (HFpEF) initiating tirzepatide versus dulaglutide, irrespective of diabetes status. This analysis offers the first real‐world head‐to‐head comparison of these agents in HFpEF, leveraging SURPASS‐CVOT insights to guide therapy in this vulnerable population.

## 2 Materials and Methods

### 2.1 Study Design and Data Source

We conducted a large multicenter, retrospective, observational, active‐comparator, cohort study using the TriNetX US Collaborative Network (Cambridge, MA, USA), a federated database aggregating de‐identified electronic health records (EHRs) from 69 healthcare organisations (HCOs) in the United States as of December 2025. The platform provides access to real‐time data on diagnoses, procedures, medications, laboratory values, and vital signs, while maintaining patient privacy through cryptographic hashing and full compliance with the Health Insurance Portability and Accountability Act (HIPAA) and General Data Protection Regulation (GDPR). All analyses utilized aggregated, de‐identified data, obviating the need for institutional review board approval. The analysis was performed on December 20, 2025, within the TriNetX Analytics suite. This study followed the Strengthening the Reporting of Observational Studies in Epidemiology (STROBE) reporting guidelines.

### 2.2 Study Population

Adults aged ≥ 18 years with at least one recorded diagnosis of heart failure with preserved ejection fraction (HFpEF) approximated by ICD‐10‐CM code I50.3 (‘Diastolic (congestive) heart failure’) at any time in their EHR were eligible. Patients were required to have a recorded prescription or administration of either tirzepatide (RxNorm code 2601723) or dulaglutide (RxNorm code 1551291), with the date of the first such record serving as the index date and proxy for treatment initiation, irrespective of diabetes status. To create clean new‐user cohorts and minimise confounding by prior exposure, patients with any recorded use of the comparator agent (dulaglutide in the tirzepatide cohort or tirzepatide in the dulaglutide cohort) were excluded. No minimum period of continuous EHR activity prior to the index date was mandated in this analysis, though index events were restricted to those occurring within the past 20 years.

### 2.3 Outcome Measures

Outcomes were assessed during a 1‐year and 3‐year time window starting 1 day after the index date and ending 365 days after the index date. All outcomes were analysed using Kaplan–Meier survival analysis, including patients with the outcome prior to the time window. All‐cause mortality was ascertained via TriNetX‐integrated linkage to death registries. Acute heart failure events were defined by ICD‐10‐CM codes I50.21, I50.23, I50.31, I50.33, I50.41, I50.43, I50.811, and I50.813. All‐cause hospitalization was defined by a comprehensive set of CPT and HCPCS codes and SNOMED terms capturing inpatient admission, observation services, and discharge management (including CPT:1013659, 1013682, 1013683, 99239, HCPCS:G0378, CPT:1013648, 1013675, 99235, 99234, 99236, and SNOMED:32485007). Myocardial infarction was defined by ICD‐10‐CM codes I21, and I22. Stroke was defined by ICD‐10‐CM codes I60–I69 (cerebrovascular diseases). Hypoglycaemia was defined by ICD‐10‐CM code E16.2. Atrial fibrillation was defined by ICD‐10‐CM code I48. Major adverse cardiovascular events (MACE) comprised a composite including myocardial infarction (I21, I22), nontraumatic intracranial hemorrhage (I61, I62), cerebral infarction (I63), and cardiac arrest (I46.2, I46.9). Major adverse kidney events (MAKE) were defined by end‐stage renal disease or kidney failure (N18.6, N18.5, N19), dependence on renal dialysis (Z99.2), and dialysis procedures (CPT:1012740, 1029674). Acute pancreatitis was defined by ICD‐10‐CM code K85 (acute pancreatitis).

### 2.4 Covariates and Propensity Score Matching

Baseline covariates were assessed in the 365‐day period preceding and including the index date and included age, sex, race/ethnicity, most recent body mass index, comorbidities (hypertension, type 2 diabetes mellitus, ischaemic heart disease, atrial fibrillation/flutter, chronic kidney disease, COPD, obesity, metabolic diseases), concomitant medications (diuretics, beta‐blockers, ACEI/ARB, insulin), and laboratory/echocardiographic parameters (systolic and diastolic blood pressure, sodium, potassium, creatinine/eGFR, haemoglobin, NT‐proBNP, LDL‐cholesterol, and left ventricular ejection fraction). Propensity scores were estimated using multivariable logistic regression incorporating all covariates listed above. One‐to‐one greedy nearest‐neighbour matching without replacement was performed using a caliper width of 0.1 on the propensity score scale. Post‐matching balance was assessed using standardised mean differences (SMD); an SMD < 0.10 for all covariates was required and confirmed. After propensity score matching, the two cohorts (tirzepatide vs. dulaglutide) were virtually identical across all measured baseline characteristics, confirming excellent covariate balance and minimising measured confounding.

### 2.5 Statistical Analysis

Time‐to‐event outcomes were analyzed using the Kaplan–Meier method, with survival curves compared between cohorts using the log‐rank test. Patients were censored at the date of their last recorded fact in the electronic health record or at the end of the follow‐up period, whichever occurred first. Hazard ratios (HRs) and corresponding 95% confidence intervals (CIs) were estimated using Cox proportional hazards regression models, with the dulaglutide cohort as the reference group. The proportional hazards assumption was assessed graphically and through Schoenfeld residuals, with associated proportionality test p‐values reported where relevant. Primary analyses were performed in the overall propensity score‐matched cohort at both 1‐year and 3‐year follow‐up. In addition, a prespecified sensitivity analysis was conducted in a restricted cohort of patients with a diagnosis of diastolic (congestive) heart failure (ICD‐10‐CM I50.3) and and LVEF ≥ 50%, based on the most recent lab measurement. Multivariable Cox proportional hazards models were also fitted to further adjust for key prognostic covariates and to evaluate the independent association of treatment with clinical outcomes. All analyses were conducted within the TriNetX Analytics platform using its built‐in statistical algorithms. A two‐tailed p‐value of less than 0.05 was considered statistically significant. No data imputation was performed, as only observed records were analyzed.

## 3 Results

### 3.1 Cohort Selection and Propensity Score Matching

**Figure 1:**
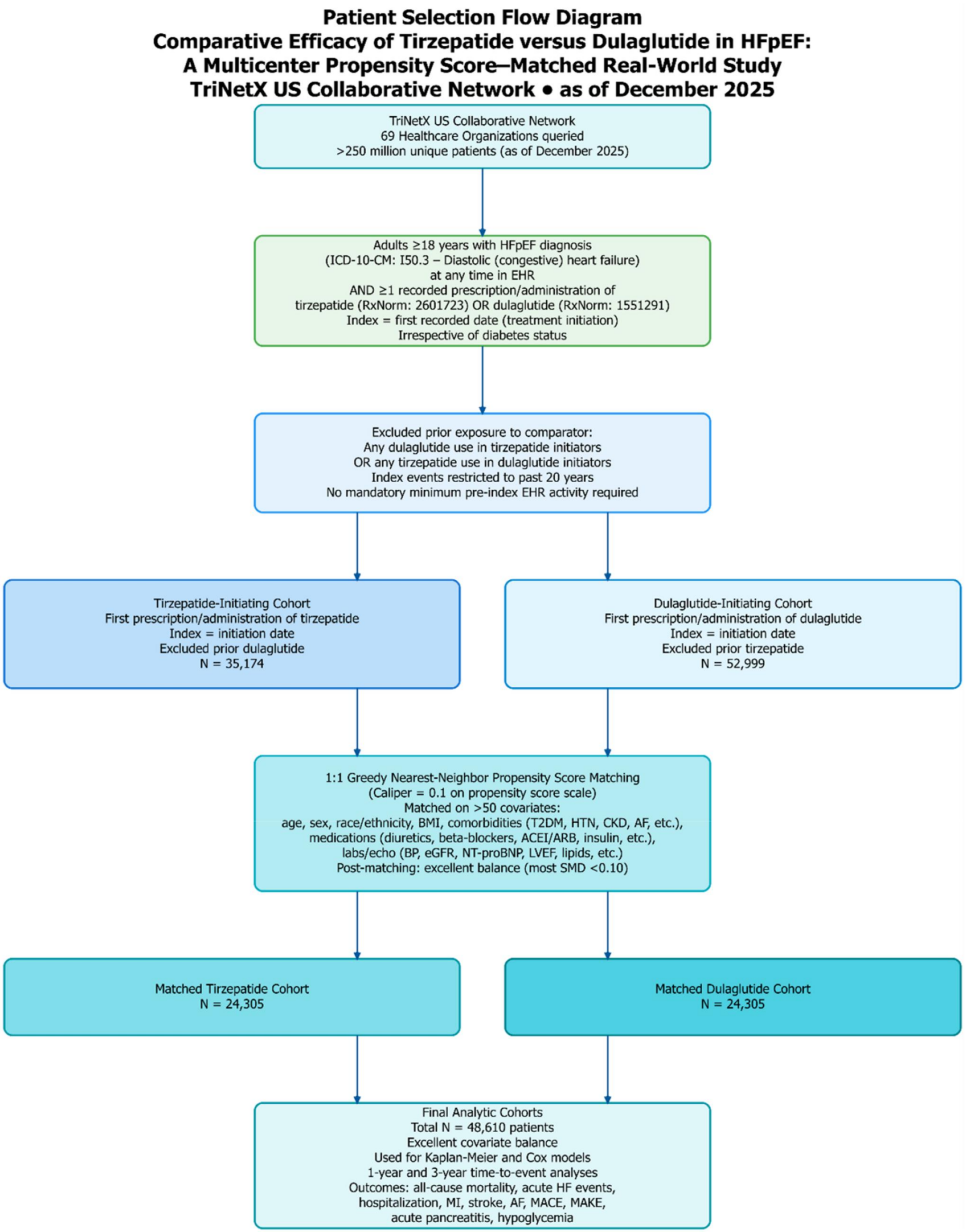
Patient selection flow diagram: Tirzepatide versus Dulaglutide in Heart Failure with Preserved Ejection Fraction (HFpEF).

This retrospective study identified 35 174 patients initiating tirzepatide and 52 999 initiating dulaglutide with HFpEF from the TriNetX US Collaborative Network, regardless of diabetes status. Pre‐matching, substantial imbalances existed: tirzepatide patients were younger (64.7 vs 69.2 years; SMD 0.390), more often female (54.8% vs 50.3%), had lower type 2 diabetes prevalence (57.4% vs 79.1%; SMD 0.479), higher BMI (39.9 vs 36.4 kg/m^2^; SMD 0.416), and differed in comorbidities, race, medications, and laboratory/echocardiographic parameters (many SMDs > 0.1; all *p* < 0.0001) (Figure 1).

**Figure 2:**
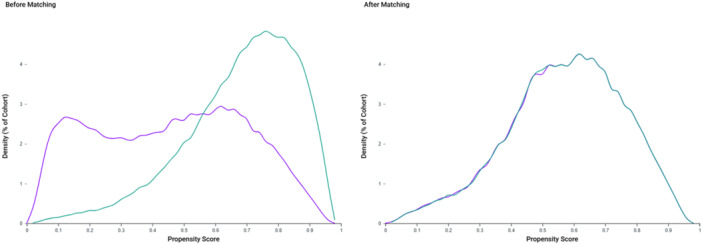
Propensity score density distributions before and after 1:1 propensity score matching in patients with heart failure with preserved ejection fraction (HFpEF). The left panel shows the distribution of propensity scores in the tirzepatide cohort (purple) and dulaglutide cohort (teal) prior to matching, demonstrating substantial overlap but with noticeable shifts reflecting baseline differences. The right panel illustrates the distributions after matching (*n* = 24 305 per cohort), with near‐complete overlap between the two cohorts, confirming excellent post‐matching balance and successful minimization of measured confounding.

**Figure 3:**
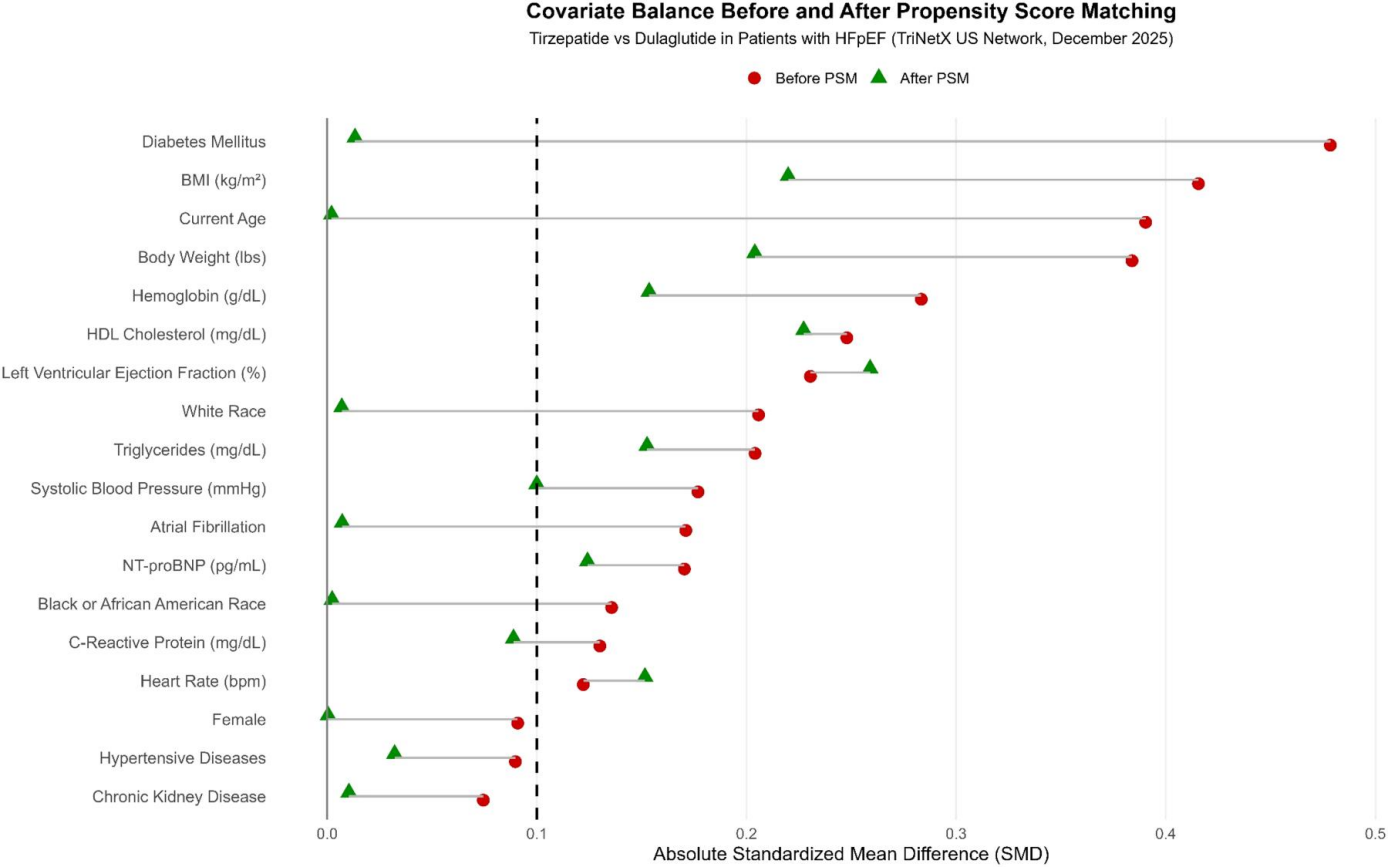
Love plot demonstrating covariate balance before and after 1:1 propensity score matching in patients with heart failure with preserved ejection fraction (HFpEF) initiating tirzepatide versus dulaglutide. Absolute standardized mean differences (SMDs) are shown for selected baseline characteristics. Red circles represent imbalances before matching; green triangles represent imbalances after matching. Dashed vertical line indicates the commonly accepted threshold for good balance (SMD = 0.1). Excellent balance was achieved for most covariates after propensity score matching, with minor residual differences in body mass index, body weight, HDL cholesterol, triglycerides, and left ventricular ejection fraction.

One‐to‐one greedy nearest‐neighbor propensity score matching (caliper 0.1) on demographics, comorbidities, medications, BMI, and laboratory/echocardiographic variables yielded balanced cohorts of 24 305 patients each. Post‐matching, excellent balance was achieved (most SMDs < 0.10; *p* > 0.05 for nearly all variables). Mean age (66.4 years both), sex (52.7% female both), race/ethnicity, and key comorbidities (e.g., diabetes ~74%–75%, atrial fibrillation ~24%) were virtually identical. Medication use aligned closely, with minor residual differences. Laboratory values improved markedly, though BMI (39.2 vs 37.3 kg/m^2^; SMD: 0.220) and HDL (43.4 vs 39.7 mg/dL; SMD: 0.227) retained slight imbalances reduced from pre‐matching levels. The propensity score density plots illustrated overlapping distributions post‐matching, supporting cohort comparability for subsequent outcome analyses (Figures 2 and 3).

**Figure 4:**
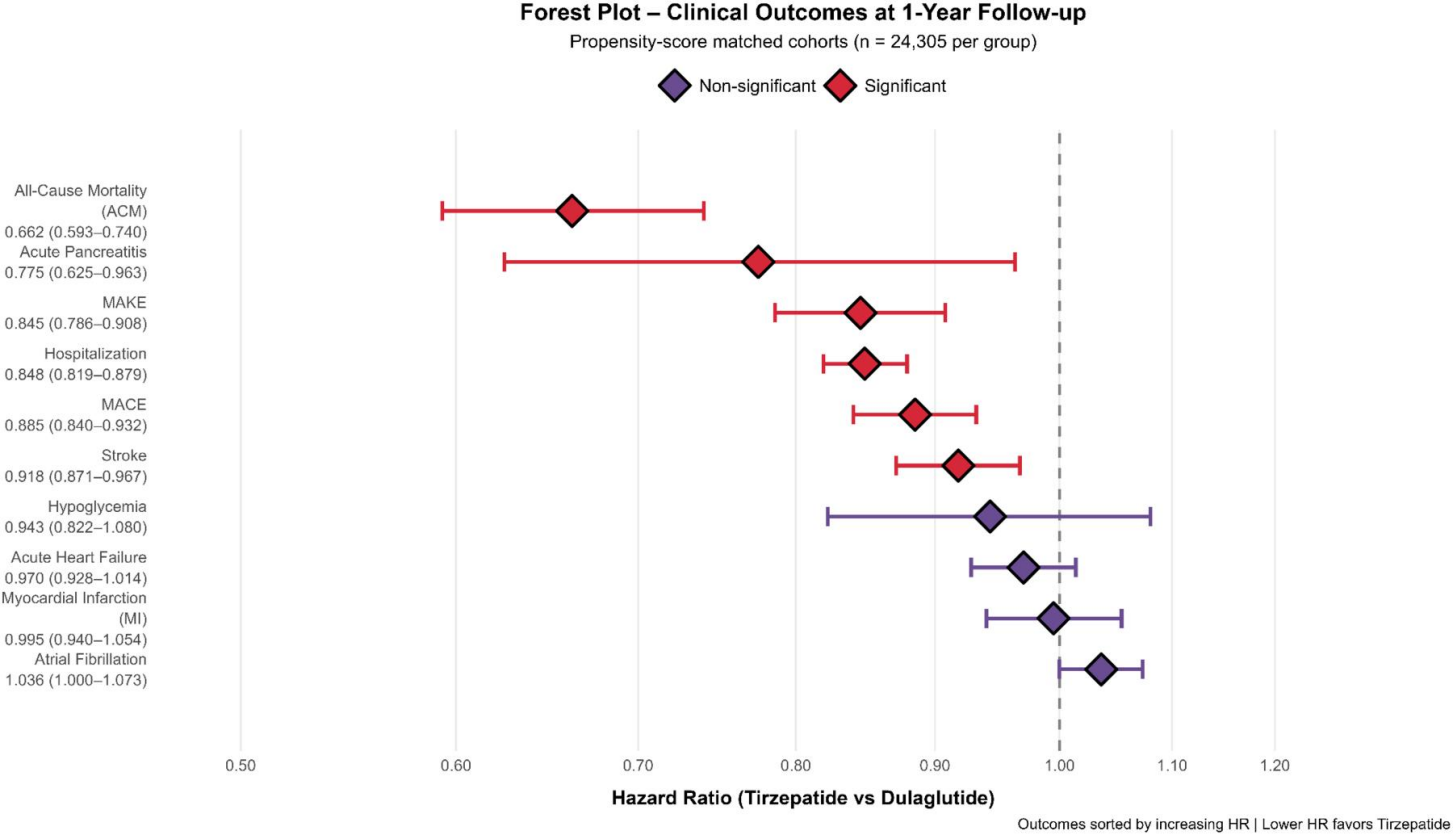
Forest plot of hazard ratios for clinical outcomes at 1‐year follow‐up in propensity score–matched cohorts of patients with heart failure with preserved ejection fraction (HFpEF) initiating tirzepatide versus dulaglutide (*n* = 24 305 per group). Hazard ratios (HRs) with 95% confidence intervals are shown, derived from Cox proportional hazards models (dulaglutide as comparison). Square points indicate statistically significant associations (log‐rank *p* < 0.05); circles indicate non‐significant associations. An HR < 1 favors tirzepatide.

**Figure 5:**
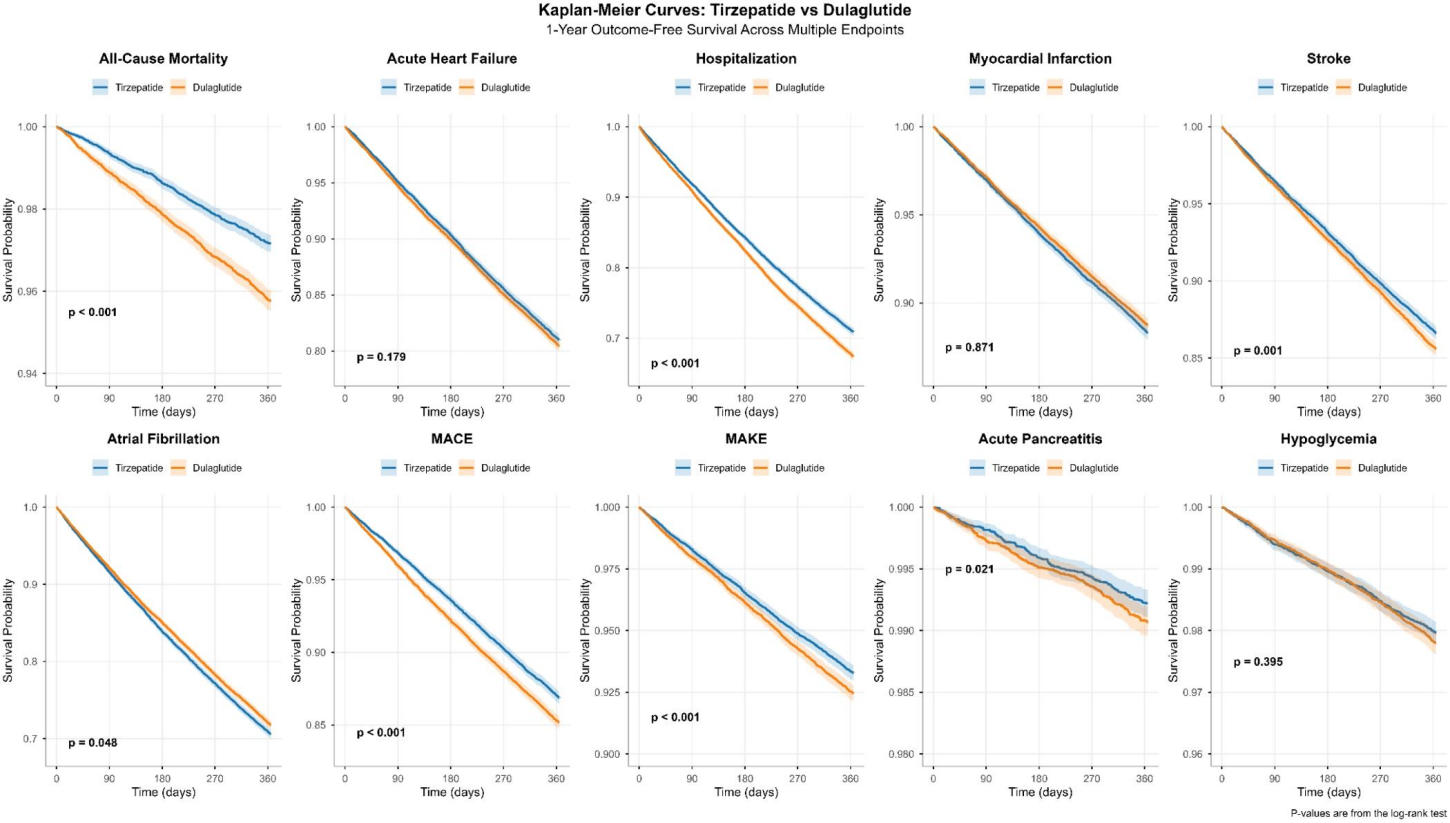
Kaplan–Meier curves for 1‐year outcome‐free survival across multiple endpoints in propensity score–matched cohorts of patients with heart failure with preserved ejection fraction (HFpEF) initiating tirzepatide (blue) versus dulaglutide (orange) (*n* = 24 305 per group). Curves represent time‐to‐first event for all‐cause mortality, acute heart failure events, all‐cause hospitalization, myocardial infarction, stroke, new‐onset atrial fibrillation, major adverse cardiovascular events (MACE), major adverse kidney events (MAKE), acute pancreatitis, and hypoglycemia. *p*‐values are derived from log‐rank tests.

**Figure 6:**
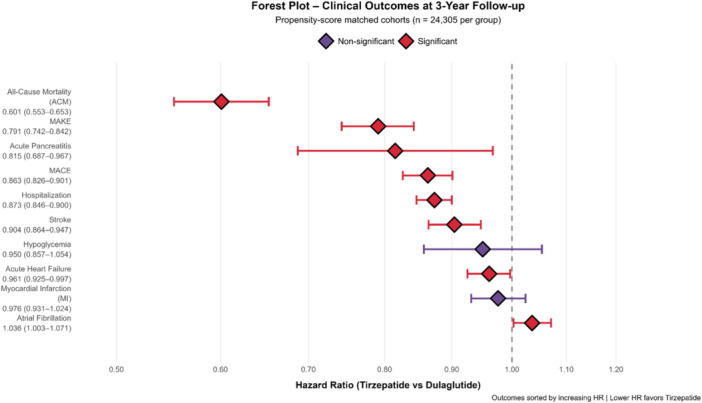
Forest plot of hazard ratios for clinical outcomes at 3‐year follow‐up in propensity score–matched cohorts of patients with heart failure with preserved ejection fraction (HFpEF) initiating tirzepatide versus dulaglutide (*n* = 24 305 per group). Hazard ratios (HRs) with 95% confidence intervals are shown, derived from Cox proportional hazards models (dulaglutide as comparison). Square points indicate statistically significant associations (log‐rank *p* < 0.05); circles indicate non‐significant associations. An HR < 1 favors tirzepatide.

**Figure 7:**
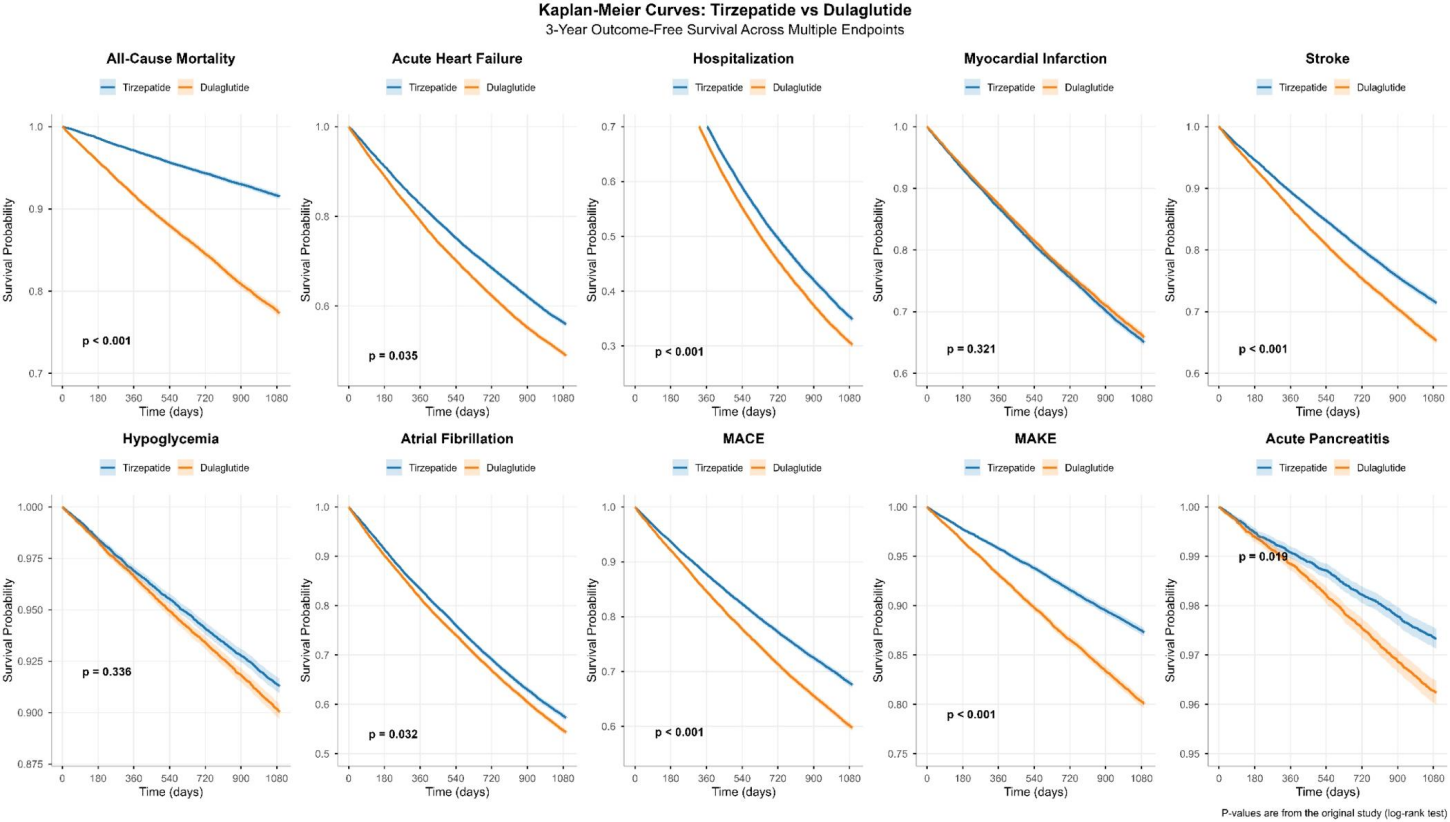
Kaplan‐Meier curves for 3‐year outcome‐free survival across multiple endpoints in propensity score–matched cohorts of patients with heart failure with preserved ejection fraction (HFpEF) initiating tirzepatide (blue) versus dulaglutide (orange) (*n* = 24 305 per group). Curves represent time‐to‐first event for all‐cause mortality, acute heart failure events, all‐cause hospitalization, myocardial infarction, stroke, new‐onset atrial fibrillation, major adverse cardiovascular events (MACE), major adverse kidney events (MAKE), acute pancreatitis, and hypoglycemia. *p*‐values are derived from log‐rank tests.

After propensity score matching, the study included 24 305 patients in the tirzepatide cohort and 24 305 patients in the dulaglutide cohort, with baseline characteristics well balanced across most of the measured covariates (detailed baseline data presented in Tables 1 and S1, Figure S1). Kaplan–Meier survival analyses were conducted for each outcome at both 1‐year and 3‐year follow‐up periods, revealing consistent patterns of benefit favoring tirzepatide in most endpoints (Figures 4, 5, 6, 7). Event counts, event free survival probabilities, hazard ratios (HRs) with 95% confidence intervals (CIs), and log‐rank P‐values are summarized below for each outcome, with comparisons highlighting the temporal evolution of treatment effects from 1 year to 3 years (Tables 2 and 3).

**Table 1:** Baseline clinical characteristics of the patients: Tirzepatide versus Dulaglutide in Heart Failure with Preserved Ejection Fraction (HFpEF).

Characteristic name	Before propensity score‐matching						After propensity score‐matched patients					
	Tirzepatide cohort (*n* = 35 174)		Dulaglutide cohort (*n* = 52 999)		*p* value	Standardized mean difference (SMD)	Tirzepatide cohort (*n* = 24 305)		Dulaglutide cohort (*n* = 24 305)		*p* value	Standardized mean difference (SMD)
Current Age, mean (SD)	64.723	11.641	69.188	11.225	< 0.0001	0.3904	66.423	10.741	66.447	11.760	0.8209	0.0021
Female, *n*(%)	19 273	54.79%	26 637	50.26%	< 0.0001	0.0909	12 804	52.68%	12810	52.71%	0.9565	0.0005
Male, *n*(%)	15 890	45.18%	26 320	49.66%	< 0.0001	0.0899	11 491	47.28%	11483	47.25%	0.9421	0.0007
BMI, kg/m2, mean (SD)	39.925	8.606	36.350	8.593	< 0.0001	0.4156	39.234	8.500	37.331	8.807	< 0.001	0.2199
Body weight, lbs, mean (SD)	251.741	60.591	228.661	59.637	< 0.0001	0.3839	247.332	59.582	235.028	61.045	< 0.001	0.2040
Ethnicity, *n*(%)												
Hispanic or Latino	1833	5.21%	4167	7.86%	< 0.0001	0.1074	1437	5.91%	1464	6.02%	0.6052	0.0047
Not Hispanic or Latino	28 024	79.67%	39 526	74.58%	< 0.0001	0.1215	18 811	77.40%	18 633	76.66%	0.0549	0.0174
Race, *n*(%)												
White	25 846	73.48%	33 913	63.99%	< 0.0001	0.2058	17 070	70.23%	16992	69.91%	0.4398	0.0070
Black or African American	6559	18.65%	12 828	24.20%	< 0.0001	0.1357	5003	20.58%	5027	20.68%	0.7879	0.0024
Asian	479	1.36%	1259	2.38%	< 0.0001	0.0749	401	1.65%	416	1.71%	0.5966	0.0048
Native Hawaiian or Other Pacific Islander	261	0.74%	451	0.85%	0.0768	0.0123	212	0.87%	220	0.91%	0.6990	0.0035
American Indian or Alaska Native	156	0.44%	278	0.53%	0.0923	0.0117	119	0.49%	122	0.50%	0.8464	0.0018
Unknown Race	992	2.82%	2178	4.11%	< 0.0001	0.0705	782	3.22%	791	3.25%	0.8176	0.0021
Diagnosis, *n*(%)												
Diabetes mellitus	20 194	57.41%	41 910	79.08%	< 0.0001	0.4785	18 081	74.39%	18222	74.97%	0.1414	0.0133
Hypertensive diseases	27 905	79.33%	40 059	75.58%	< 0.0001	0.0898	18 706	76.96%	19032	78.31%	0.0004	0.0322
Metabolic disorders	25 880	73.58%	36 685	69.22%	< 0.0001	0.0966	17 388	71.54%	17632	72.55%	0.0137	0.0224
Ischemic heart diseases	14 036	39.90%	20 962	39.55%	0.2944	0.0072	9825	40.42%	9998	41.14%	0.1103	0.0145
Chronic kidney disease (CKD)	10 297	29.27%	17 340	32.72%	< 0.0001	0.0745	7763	31.94%	7881	32.43%	0.2520	0.0104
Atrial fibrillation and flutter	9729	27.66%	10 799	20.38%	< 0.0001	0.1711	5907	24.30%	5982	24.61%	0.4287	0.0072
Acute kidney failure	4687	13.33%	9026	17.03%	< 0.0001	0.1034	3640	14.98%	3694	15.20%	0.4938	0.0062
Cardiomyopathy	5619	15.98%	6482	12.23%	< 0.0001	0.1077	3401	13.99%	3388	13.94%	0.8649	0.0015
Pulmonary heart disease and diseases of pulmonary circulation	5682	16.15%	6032	11.38%	< 0.0001	0.1388	3383	13.92%	3412	14.04%	0.7045	0.0034
Cerebrovascular diseases	3623	10.30%	6575	12.41%	< 0.0001	0.0664	2702	11.12%	2752	11.32%	0.4724	0.0065
Chronic rheumatic heart diseases	2826	8.03%	3208	6.05%	< 0.0001	0.0775	1767	7.27%	1784	7.34%	0.7670	0.0027
Medications, *n*(%)												
Anti‐lipemic agents	20 832	59.23%	32 903	62.08%	< 0.0001	0.0585	14 770	60.77%	15029	61.84%	0.0159	0.0219
Diuretics	21 519	61.18%	29 220	55.13%	< 0.0001	0.1228	14 069	57.89%	14207	58.45%	0.2045	0.0115
Insulin	11 198	31.84%	28 940	54.61%	< 0.0001	0.4723	10 314	42.44%	10594	43.59%	0.0103	0.0233
Beta‐ blockers/Related	19 763	56.19%	28 419	53.62%	< 0.0001	0.0516	13 237	54.46%	13418	55.21%	0.0990	0.0150
Oral hypoglycemic agents	15 587	44.31%	28 663	54.08%	< 0.0001	0.1963	12 355	50.83%	12551	51.64%	0.0753	0.0161
Anti‐arrhythmatics	15 488	44.03%	19 223	36.27%	< 0.0001	0.1588	9839	40.48%	9843	40.50%	0.9705	0.0003
Calcium channel blockers	11 273	32.05%	17 581	33.17%	0.0005	0.0240	7807	32.12%	8014	32.97%	0.0451	0.0182
ACE Inhibitors	6042	17.18%	14130	26.66%	< 0.0001	0.2308	4842	19.92%	4932	20.29%	0.3085	0.0092
Angiotensin II Inhibitors	13 114	37.28%	15 092	28.48%	< 0.0001	0.1883	8238	33.89%	8239	33.90%	0.9924	0.0001
Antianginals	6051	17.20%	10 612	20.02%	< 0.0001	0.0725	4398	18.10%	4510	18.56%	0.1892	0.0119
Digitalis glycosides	641	1.82%	1243	2.35%	< 0.0001	0.0366	478	1.97%	519	2.14%	0.1895	0.0119
Peripheral vasodilators	262	0.75%	492	0.93%	0.0038	0.0201	218	0.90%	211	0.87%	0.7343	0.0031
Direct renin inhibitors	10	0.03%	10	0.02%	0.3559	0.0062	10	0.04%	10	0.04%	1.0000	< 0.0001
SGLT2 inhibitors	6099	17.34%	10 505	19.82%	< 0.0001	0.1071	3986	16.4%	3913	16.1%	0.0622	0.0137
Laboratory Values, Mean (SD)												
Creatinine, mEq/L, mean (SD)	1.366	4.363	1.456	3.725	0.0039	0.0222	1.457	4.930	1.486	4.499	0.5480	0.0062
Sodium, mEq/L, mean (SD)	139.130	2.911	138.253	3.326	< 0.0001	0.2806	138.991	2.991	138.305	3.260	< 0.001	0.2191
Potassium, mEq/L, mean (SD)	4.255	0.474	4.295	0.507	< 0.0001	0.0833	4.277	0.479	4.282	0.506	0.2673	0.0114
Heart rate, bpm, mean (SD)	77.288	14.360	79.044	14.378	< 0.0001	0.1222	77.374	14.149	79.545	14.496	< 0.001	0.1516
Systolic blood pressure, mmHg, mean (SD)	128.366	19.798	131.908	20.240	< 0.0001	0.1769	129.045	19.988	131.042	19.956	< 0.001	0.1000
Diastolic blood pressure, mmHg, mean (SD)	75.165	13.276	73.585	12.010	< 0.0001	0.1248	74.345	12.952	74.045	12.170	0.0210	0.0239
Hemoglobin, g/dL, mean (SD)	13.169	1.957	12.594	2.096	< 0.0001	0.2834	13.026	1.993	12.711	2.109	< 0.001	0.1536
HDL cholesterol, mg/dL, mean (SD)	44.174	16.617	40.151	15.830	< 0.0001	0.2479	43.382	15.833	39.738	16.222	< 0.001	0.2273
Cholesterol, mg/dL, mean (SD)	157.238	44.146	157.807	49.654	0.1953	0.0121	154.139	44.414	158.651	48.742	< 0.001	0.0968
Triglyceride, mg/dL, mean (SD)	155.448	118.392	183.667	155.586	< 0.0001	0.2041	161.721	126.696	182.950	150.479	< 0.001	0.1526
LDL cholesterol, mg/dL, mean (SD)	82.711	36.409	81.491	38.455	0.0004	0.0326	79.667	36.106	82.394	38.366	< 0.001	0.0732
NT‐proBNP, pg/mL, mean (SD)	1514.233	4535.960	2375.014	5521.542	< 0.0001	0.1704	1659.047	4596.586	2298.283	5641.351	< 0.001	0.1242
*C* reactive protein, mg/dL, mean (SD)	29.348	52.371	36.558	58.316	< 0.0001	0.1301	30.921	54.617	35.883	56.944	0.0008	0.0889
Troponin I, ng/mL, mean (SD)	1.248	19.494	0.785	6.362	0.1855	0.0319	1.332	20.893	0.463	3.427	0.1934	0.0580
Left ventricular ejection fraction (LVEF) (%), mean (SD)	56.786	11.982	53.768	14.119	< 0.0001	0.2305	57.012	12.063	53.581	14.336	< 0.001	0.2590

**Table 2:** Table showing the Kaplan–Meier survival analyses for all clinical outcomes comparing Tirzepatide versus Dulaglutide after propensity score matching (*n* = 24 305 per cohort) at 1‐Year follow‐up.

	Outcome	Tirzepatide cohort events (*n* = 24 305)	Dulaglutide cohort events (*n* = 24 305)	Tirzepatide cohort KM event‐free survival probability (%)	Dulaglutide cohort KM event‐free survival probability (%)	Hazard ratio (95% CI)	Log‐Rank *χ* ^2^(df = 1)	Log‐Rank *p* value
1	All‐cause mortality (ACM)	475	952	97.15	95.80	0.662 (0.593–0.740)	54.195	< 0.001
2	Acute heart failure	3522	4431	81.01	80.70	0.970 (0.928–1.014)	1.807	0.179
3	All‐cause hospitalization	5329	7428	71.28	67.90	0.848 (0.819–0.879)	83.337	< 0.001
4	myocardial infarction (MI)	2136	2,658	88.27	88.33	0.995 (0.940–1.054)	0.027	0.871
5	Stroke	2464	3242	86.83	85.84	0.918 (0.871–0.967)	10.293	0.001
6	Atrial fibrillation	5939	6534	70.56	72.03	1.036 (1.000–1.073)	3.913	0.048
7	MACE	2483	3404	86.62	85.15	0.885 (0.840–0.932)	21.332	< 0.001
8	MAKE	1293	1801	93.34	92.20	0.845 (0.786–0.908)	21.285	< 0.001
9	Acute pancreatitis	134	216	99.25	99.05	0.775 (0.625–0.963)	5.345	0.021
10	Hypoglycemia	361	491	97.92	97.82	0.943 (0.822–1.080)	0.724	0.395

**Table 3:** Table showing the Kaplan–Meier survival analyses for all clinical outcomes comparing Tirzepatide versus Dulaglutide after propensity score matching (*n* = 24 305 per cohort) at 3‐Year follow‐up.

	Outcome	Tirzepatide cohort events (*n* = 24 305)	Dulaglutide cohort events (*n* = 24 305)	Tirzepatide cohort KM event‐free survival probability (%)	Dulaglutide cohort KM event‐free survival probability (%)	Hazard ratio (95% CI)	Log‐Rank *χ* ^2^(df = 1)	Log‐Rank *p* value
1	All‐cause mortality (ACM)	742	3492	91.42	77.03	0.601 (0.553–0.653)	146.824	<0.001
2	Acute heart failure	4563	8972	56.24	49.45	0.961 (0.925–0.997)	4.444	0.035
3	All‐cause hospitalization	6796	13 168	34.73	30.63	0.873 (0.846–0.900)	76.792	<0.001
4	Myocardial infarction (MI)	2821	5777	65.07	65.47	0.976 (0.931–1.024)	0.984	0.321
5	Stroke	3059	6006	71.86	65.59	0.904 (0.864–0.947)	18.749	<0.001
6	Atrial fibrillation	6593	9074	56.58	53.96	1.036 (1.003–1.071)	4.597	0.032
7	MACE	3194	6999	67.52	59.48	0.863 (0.826–0.901)	43.601	<0.001
8	MAKE	1530	3439	87.25	79.84	0.791 (0.742–0.842)	53.603	<0.001
9	Acute pancreatitis	199	557	97.29	96.33	0.815 (0.687–0.967)	5.542	0.019
10	Hypoglycemia	559	1438	91.45	90.25	0.950 (0.857–1.054)	0.927	0.336

### 3.2 All‐Cause Mortality (ACM)

Tirzepatide demonstrated a substantial and sustained reduction in all‐cause mortality compared to dulaglutide across both short‐ and long‐term follow‐up (Figures 4, 5, 6, 7). At 1 year, 475 events occurred in the tirzepatide group versus 952 in the dulaglutide group, resulting in KM event free survival probabilities of 97.15% and 95.80%, respectively (HR 0.662; 95% CI: 0.593–0.740; log‐rank *χ* ^2^ 54.195; *p* < 0.001). This early separation in Kaplan‐Meier curves indicated a 33.8% relative risk reduction, potentially attributable to tirzepatide's dual incretin agonism enhancing metabolic control, weight loss, and anti‐inflammatory effects in the HFpEF population. By 3 years, the benefit amplified, with 742 events in the tirzepatide cohort compared to 3492 in the dulaglutide cohort, yielding survival rates of 91.42% versus 77.03% (HR: 0.601; 95% CI: 0.553–0.653; log‐rank *χ* ^2^ 146.824; *p* < 0.001). The widening gap over time suggests cumulative protective effects on cardiovascular and renal comorbidities common in HFpEF, with an absolute risk reduction increasing from approximately 1.35% at 1 year to 14.39% at 3 years, underscoring tirzepatide's potential for long‐term mortality benefit in this high‐risk group.

### 3.3 Acute Heart Failure Events

The impact of tirzepatide on acute heart failure events showed a modest but progressively significant advantage over dulaglutide when extending from 1‐year to 3‐year follow‐up (Figures 4, 5, 6, 7). In the first year, there were 3522 events in the tirzepatide group and 4431 in the dulaglutide group, with event‐free survival rates of 81.01% and 80.70%, respectively (HR: 0.970; 95% CI: 0.928–1.014; log‐rank *χ* ^2^: 1.807; *p* = 0.179), indicating no statistically significant difference initially, possibly due to the early phase where weight loss and hemodynamic improvements are still emerging. However, over 3 years, the divergence became apparent, with 4563 events for tirzepatide versus 8972 for dulaglutide, leading to event free survival probabilities of 56.24% and 49.45% (HR: 0.961; 95% CI: 0.925–0.997; log‐rank *χ* ^2^: 4.444; *p* = 0.035). This delayed benefit, representing a 3.9% relative risk reduction, may reflect tirzepatide's superior effects on epicardial fat reduction, diastolic function, and natriuresis, which accumulate to prevent recurrent decompensations in HFpEF patients over longer durations.

### 3.4 All‐Cause Hospitalization

Tirzepatide consistently reduced the risk of all‐cause hospitalization compared to dulaglutide, with benefits evident early and persisting through extended follow‐up (Figures 4, 5, 6, 7). At 1 year, hospitalization events numbered 5329 in the tirzepatide cohort and 7428 in the dulaglutide cohort, translating to hospitalization‐free survival of 71.28% versus 67.90% (HR: 0.848; 95% CI: 0.819–0.879; log‐rank *χ* ^2^: 83.337; *p* < 0.001), highlighting a 15.2% relative risk reduction that could stem from better glycemic control, weight management, and fewer acute exacerbations of comorbidities. Extending to 3 years, the pattern held with 6796 events for tirzepatide and 13 168 for dulaglutide, resulting in survival rates of 34.73% and 30.63% (HR: 0.873; 95% CI: 0.846–0.900; log‐rank *χ* ^2^: 76.792; *p* < 0.001). Although the relative reduction slightly attenuated to 12.7%, the absolute difference grew from 3.38% to 4.10%, emphasizing tirzepatide's role in mitigating the cumulative burden of multimorbidity in HFpEF, potentially leading to fewer unplanned admissions over time.

### 3.5 Major Adverse Cardiovascular Events (MACE)

Tirzepatide significantly lowered the composite risk of major adverse cardiovascular events (MACE) compared to dulaglutide, with pronounced effects at both 1 and 3 years (Figures 4, 5, 6, 7). In the first year, MACE events were 2483 versus 3404, leading to event‐free survival of 86.62% and 85.15% (HR: 0.885; 95% CI: 0.840–0.932; log‐rank *χ* ^2^: 21.332; *p* < 0.001), reflecting an 11.5% relative reduction driven by components like stroke and cardiac arrest. Extending to 3 years, events rose to 3194 and 6999, with event free survival probabilities of 67.52% versus 59.48% (HR: 0.863; 95% CI: 0.826–0.901; log‐rank *χ* ^2^: 43.601; *p* < 0.001). The enhanced 13.7% relative risk reduction over longer follow‐up underscores tirzepatide's multifaceted cardioprotection, including superior inflammation modulation and metabolic effects, resulting in a cumulative absolute benefit increasing from 1.47% to 8.04% in this HFpEF population.

### 3.6 Major Adverse Kidney Events (MAKE)

The renoprotective effects of tirzepatide were evident in reduced major adverse kidney events (MAKE) relative to dulaglutide, with benefits apparent early and intensifying over time (Figures 4, 5, 6, 7). At 1 year, 1293 events occurred with tirzepatide compared to 1801 with dulaglutide, yielding MAKE‐free survival of 93.34% versus 92.20% (HR: 0.845; 95% CI: 0.786–0.908; log‐rank *χ* ^2^: 21.285; *p* < 0.001), a 15.5% relative reduction possibly linked to better glomerular filtration preservation through weight loss and anti‐fibrotic actions. By 3 years, events were 1530 versus 3439, with survival rates of 87.25% and 79.84% (HR: 0.791; 95% CI: 0.742–0.842; log‐rank *χ* ^2^: 53.603; *p* < 0.001). The greater 20.9% relative risk reduction highlights tirzepatide's long‐term efficacy in delaying progression to end‐stage renal disease or dialysis in HFpEF patients with overlapping cardiorenal risks, with absolute differences expanding from 1.14% to 7.41%.

### 3.7 Myocardial Infarction (MI)

No significant differences were observed between tirzepatide and dulaglutide in the risk of myocardial infarction at either 1‐year or 3‐year follow‐up, suggesting comparable effects on coronary events in this HFpEF cohort (Figures 4, 5, 6, 7). In the first year, 2136 events occurred with tirzepatide compared to 2658 with dulaglutide, with MI event free survival probabilities of 88.27% and 88.33% (HR: 0.995; 95% CI: 0.940–1.054; log‐rank *χ* ^2^: 0.027; *p* = 0.871), indicating near‐equivalence possibly due to shared GLP‐1 receptor‐mediated anti‐atherosclerotic mechanisms. Over 3 years, events increased to 2821 versus 5777, yet survival rates remained similar at 65.07% and 65.47% (HR: 0.976; 95% CI: 0.931–1.024; log‐rank *χ* ^2^: 0.984; *p* = 0.321). The lack of divergence over time may reflect the predominance of microvascular rather than macrovascular pathology in HFpEF, where tirzepatide's additional GIP agonism does not confer extra protection against MI beyond dulaglutide's established benefits.

### 3.8 Stroke

Tirzepatide was associated with a reduced risk of stroke relative to dulaglutide, with benefits manifesting at 1 year and strengthening over 3 years (Figures 4, 5, 6, 7). At the 1‐year mark, stroke events were 2464 in the tirzepatide group and 3242 in the dulaglutide group, yielding stroke‐free survival of 86.83% versus 85.84% (HR: 0.918; 95% CI: 0.871–0.967; log‐rank *χ* ^2^: 10.293; *p* = 0.001), a 8.2% relative reduction that aligns with tirzepatide's enhanced endothelial protection and anti‐inflammatory properties. By 3 years, the advantage persisted with 3,059 events versus 6006, resulting in event free survival probabilities of 71.86% and 65.59% (HR: 0.904; 95% CI: 0.864–0.947; log‐rank *χ* ^2^: 18.749; *p* < 0.001). The sustained 9.6% relative risk reduction, with an increasing absolute benefit from 0.99% to 6.27%, highlights tirzepatide's potential superiority in preventing cerebrovascular events, possibly through greater weight loss and lipid improvements in the context of HFpEF ‘s inflammatory milieu.

### 3.9 Atrial Fibrillation

In contrast to other outcomes, tirzepatide was linked to a slightly higher risk of atrial fibrillation compared to dulaglutide, though the differences were marginal and consistent across time frames (Figures 4, 5, 6, 7). At 1 year, events totaled 5,939 for tirzepatide and 6534 for dulaglutide, with arrhythmia‐free survival of 70.56% versus 72.03% (HR: 1.036; 95% CI: 1.000–1.073; log‐rank *χ* ^2^: 3.913; *p* = 0.048), suggesting a 3.6% relative increase that might relate to transient sympathetic activation or electrolyte shifts during rapid weight loss. Over 3 years, this pattern continued with 6593 events versus 9074, and survival rates of 56.58% and 53.96% (HR: 1.036; 95% CI: 1.003–1.071; log‐rank *χ* ^2^: 4.597; *p* = 0.032). The stable HR indicates no worsening over time, but the finding warrants caution in HFpEF patients prone to arrhythmias, potentially necessitating closer monitoring despite the overall cardiovascular benefits of tirzepatide.

### 3.10 Acute Pancreatitis

Tirzepatide was associated with a lower risk of acute pancreatitis than dulaglutide, with consistent findings across 1‐year and 3‐year periods, though event rates were low overall (Figures 4, 5, 6, 7). In the first year, 134 events were recorded for tirzepatide versus 216 for dulaglutide, resulting in pancreatitis‐free survival of 99.25% and 99.05% (HR: 0.775; 95% CI: 0.625–0.963; log‐rank *χ* ^2^: 5.345; *p* = 0.021), indicating a 22.5% relative reduction that may reflect tirzepatide's balanced receptor activation minimizing gastrointestinal side effects leading to pancreatitis. Over 3 years, events increased to 199 and 557, with event free survival probabilities of 97.29% versus 96.33% (HR: 0.815; 95% CI: 0.687–0.967; log‐rank *χ* ^2^: 5.542; *p* = 0.019). The sustained 18.5% relative benefit, despite low incidence, suggests tirzepatide's favorable safety profile in this regard, with absolute risk reductions remaining small (0.20% at 1 year to 0.96% at 3 years) but clinically relevant for long‐term therapy in multimorbid patients.

### 3.11 Hypoglycemia

No significant differences in hypoglycemia risk were found between tirzepatide and dulaglutide at either time point, reflecting the glucose‐dependent mechanisms of both agents (Figures 4, 5, 6, 7). At 1 year, events were 361 versus 491, with hypoglycemia‐free survival of 97.92% and 97.82% (HR: 0.943; 95% CI: 0.822–1.080; log‐rank *χ* ^2^: 0.724; *p* = 0.395), suggesting equivalence in this safety endpoint despite tirzepatide's greater potency. Similarly, at 3 years, 559 events occurred with tirzepatide compared to 1438 with dulaglutide, yielding survival rates of 91.45% and 90.25% (HR: 0.950; 95% CI: 0.857–1.054; log‐rank *χ* ^2^: 0.927; *p* = 0.336). The lack of divergence over time supports the safety of tirzepatide in HFpEF patients, many of whom have concomitant diabetes, without increased hypoglycemic burden relative to dulaglutide.

### 3.12 Multivariate Cox Proportional Hazards Model and Sensitivity Analysis

In the multivariate Cox proportional hazards model for all‐cause mortality, male sex was associated with a significantly higher risk (HR: 1.35, 95% CI: 1.291–1.411, *p* < 0.001), as was the presence of chronic kidney disease (CKD; HR: 2.148, 95% CI: 2.038–2.262, *p* < 0.001) and a history of acute myocardial infarction (HR: 1.765, 95% CI: 1.618–1.925, *p* < 0.001). These findings highlight the strong independent prognostic impact of male sex, renal impairment, and prior coronary events on mortality in this propensity score–matched HFpEF cohort treated with incretin‐based therapies. In contrast, categorized LVEF in the preserved range showed no significant association with mortality risk; LVEF 50%–60% (HR: 0.968, 95% CI: 0.804–1.167, *p* = 0.736) and 60%–70% (HR: 0.962, 95% CI: 0.804–1.151, *p* = 0.674) exhibited comparable hazard rates, indicating that finer gradations within the preserved ejection fraction spectrum did not meaningfully influence all‐cause death in this population after extensive covariate adjustment.

For the separate Cox model examining acute HF events, male sex conferred a modestly increased risk (HR: 1.035, 95% CI: 1.019–1.052, *p* < 0.001), while CKD (HR: 1.718, 95% CI: 1.684–1.752, *p* < 0.001) and prior acute myocardial infarction (HR: 1.799, 95% CI: 1.736–1.865, *p* < 0.001) emerged as more potent predictors of decompensation requiring admission. Notably, preserved‐range LVEF categories were slightly linked to higher HF event risk, with HRs of 1.439 (95% CI: 1.353–1.530, *p* < 0.001) for LVEF 50%–60% and 1.422 (95% CI: 1.344–1.504, *p* < 0.001) for LVEF 60%–70%, suggesting that even within the HFpEF diagnostic category, relatively lower preserved ejection fractions identify patients at substantially greater risk of recurrent acute heart failure events despite balanced baseline characteristics and treatment assignment.

To assess the robustness of the primary findings, we conducted a prespecified sensitivity analysis restricted to patients with diastolic (congestive) heart failure (ICD‐10‐CM I50.3) and LVEF ≥ 50%, based on the most recent lab measurement. In this higher‐risk subgroup with HFpEF, the results were largely consistent with the main analysis, demonstrating a stronger relative benefit for tirzepatide (Table S2). Tirzepatide was associated with a substantially lower risk of all‐cause mortality (2.17% vs 5.60%; HR: 0.465, 95% CI: 0.364–0.593, *p* < 0.001), representing a 53.5% relative risk reduction. Similarly, marked reductions were observed for acute heart failure (HR: 0.661, 95% CI: 0.600–0.728, *p* < 0.001), all‐cause hospitalization (HR: 0.585, 95% CI: 0.544–0.629, *p* < 0.001), myocardial infarction (HR: 0.692, 95% CI: 0.591–0.812, *p* < 0.001), MACE (HR: 0.794, 95% CI: 0.707–0.892, *p* < 0.001), and MAKE (HR: 0.557, 95% CI: 0.469–0.660, *p* < 0.001). Notably, the magnitude of risk reduction for all‐cause mortality and major adverse kidney events (MAKE) was greater in this sensitivity cohort compared with the main analysis, where the corresponding HRs were 0.662 and 0.845, respectively. These findings suggest that the cardiovascular and renal protective effects of tirzepatide versus dulaglutide may be even more pronounced.

## 4 Discussion

### 4.1 Principal Findings and Clinical Significance

In this large propensity score–matched real‐world cohort of 48 610 patients with HFpEF, tirzepatide initiation was associated with significantly lower risks of all‐cause mortality (1‐year HR: 0.662; 3‐year HR: 0.601), acute heart failure events (3‐year HR: 0.961), all‐cause hospitalization (1‐year HR: 0.848; 3‐year HR: 0.873), stroke (1‐year HR: 0.918; 3‐year HR: 0.904), major adverse cardiovascular events (MACE; 1‐year HR: 0.885; 3‐year HR: 0.863), major adverse kidney events (MAKE; 1‐year HR: 0.845; 3‐year HR: 0.791), and acute pancreatitis (1‐year HR: 0.775; 3‐year HR: 0.815) compared with dulaglutide (Figures 4, 5, 6, 7). Conversely, a modest increase in atrial fibrillation risk was observed (1‐year HR: 1.036; 3‐year HR: 1.036), while myocardial infarction and hypoglycemia showed no significant differences. Absolute benefits amplified over time, with survival differences widening markedly by 3 years, particularly for mortality (absolute risk reduction 14.39%) and MAKE (7.41%). Multivariate Cox proportional hazards regression and the sensitivity analysis were consistent with the main analysis, further confirming the robustness of the observed associations. These findings represent the first direct real‐world head‐to‐head comparison of tirzepatide and dulaglutide in HFpEF, highlighting tirzepatide's superior efficacy in reducing hard clinical endpoints in this multimorbid, obesity‐driven population, irrespective of diabetes status. The results extend the noninferiority trend from SURPASS‐CVOT [12] into a dedicated HFpEF context, suggesting tirzepatide's dual GLP‐1/GIP agonism confers additive benefits beyond single‐receptor GLP‐1RAs like dulaglutide.

### 4.2 Comparison With Established HFpEF Therapies

The magnitude of tirzepatide's mortality reduction of 33.8% relative at 1 year and 39.9% at 3 years, surpasses signals from cornerstone HFpEF trials. For instance, empagliflozin in EMPEROR‐Preserved reduced the composite of cardiovascular death or HF hospitalization by 21% (HR: 0.79; 95% CI: 0.69–0.90; *p* < 0.001) but failed to significantly impact all‐cause mortality (HR: 1.00; 95% CI: 0.87–1.15) [3]. Similarly, dapagliflozin in DELIVER achieved an 18% reduction in the same composite (HR: 0.82; 95% CI: 0.73–0.92) without direct cardiovascular mortality benefit (HR: 0.88; 95% CI: 0.74–1.05) or all‐cause mortality reduction (HR: 0.94; 95% CI: 0.83–1.07) [19]. Sacubitril/valsartan in PARAGON‐HF offered a non‐significant 13% composite reduction (HR: 0.87; 95% CI: 0.75–1.01; *p* = 0.06), including non‐significant reduction in cardiovascular death (HR: 0.95; 95% CI: 0.79 to 1.16), and hospitalizations for heart failure (RR: 0.85; 95% CI: 0.72 to 1.00) [20].

Spironolactone in TOPCAT reduced hospitalization for heart failure (HR: 0.83; 95% CI: 0.69–0.99, *p* = 0.04), but did not significantly reduce the primary composite outcome (HR: 0.89; 95% CI: 0.77 to 1.04; *p* = 0.14), all‐cause mortality (HR: 0.91; 95% CI: 0.77 to 1.08; *p* = 0.29) nor all‐cause hospitalizations (HR: 0.94; 95% CI: 0.85 to 1.04; *p* = 0.25) [21]. Tirzepatide's sustained 3‐year mortality advantage (ARR = 14.39%; NNT ≈ 7 for preventing one death) positions it among the most potent interventions in HFpEF pharmacotherapy, potentially rivaling or complementing SGLT2 inhibitors, emphasizing tirzepatide's role in addressing HFpEF's unmet needs beyond glycemic control.

### 4.3 Context Within the GLP‐1 Receptor Agonist Class

Our results challenge the notion of a uniform class effect among incretin‐based therapies in HFpEF, underscoring agent‐specific differences driven by molecular design and pharmacokinetics. Tirzepatide's dual agonism, with balanced GLP‐1/GIP activation, likely amplifies weight loss (SURPASS‐1, SURPASS‐2) [11, 17], inflammation attenuation, and cardiorenal protection [13, 14, 15, 16], translating to superior outcomes here. In contrast, single‐receptor GLP‐1RAs like dulaglutide, while effective in broad T2DM populations (REWIND: 12% MACE reduction) [10], appear less potent in HFpEF's obesity‐centric phenotype. Recent dedicated trials support a therapeutic hierarchy among incretin‐based therapies in obesity‐related heart failure with preserved ejection fraction (HFpEF). In the STEP‐HFpEF program, semaglutide improved heart failure symptoms, physical limitations, and exercise capacity, while demonstrating only modest reductions in heart failure hospitalizations [7, 8]. In contrast, the SUMMIT trial showed that tirzepatide achieved a more substantial reduction in the composite endpoint of cardiovascular death or worsening heart failure events (HR: 0.62; 95% CI: 0.41–0.95; *p* = 0.026), including a significant decrease in worsening heart failure events (HR: 0.54; 95% CI: 0.34–0.85). This was accompanied by superior weight loss (− 13.9% in the tirzepatide group *vs.* −2.2% in the placebo group; mean difference, −11.6%; 95% CI: −12.9 to −10.4; *p* < 0.001) [9]. However, tirzepatide did not significantly reduce all‐cause mortality (HR: 1.25; 95% CI: 0.63–2.45) nor cardiovascular mortality (HR: 1.58; 95% CI: 0.52–4.83) [9].

A 2025 meta‐analysis of GLP‐1RAs in HFpEF pooled semaglutide and tirzepatide data, revealing a 36% reduction in worsening HF or CV death (RR: 0.64, 95% CI: 0.45–0.92) [22]. Still, our direct comparison isolates tirzepatide's edge over dulaglutide. However, comparisons via a contemporaneous Bayesian network meta‐analysis provide nuanced insights into the relative efficacy of GLP‐1RAs in HFpEF [23]. For prevention of HHF, tirzepatide ranked highest (HR: 0.51; 95% CrI: 0.16–1.24; SUCRA, 76.94%), followed by semaglutide (HR: 0.52; 95% CrI: 0.25–0.87; SUCRA: 74.01%). Regarding all‐cause mortality, semaglutide (HR: 0.80; 95% CrI: 0.64–0.97; SUCRA: 71.39%) and exenatide (HR: 0.73; 95% CrI: 0.56–0.95; SUCRA: 86.86%) reduced risk relative to placebo, whereas tirzepatide showed less effect (HR: 1.25; 95% CrI: 0.60–2.31; SUCRA: 18.23%), and placebo ranked intermediately (SUCRA: 23.53%) [23]. Real‐world data from 2025 further validate this, with semaglutide (HR: 0.58, 95% CI: 0.51 to 0.65) and tirzepatide (HR: 0.42, 95% CI: 0.31 to 0.57) reducing HF hospitalization or death compared to sitagliptin in cardiometabolic HFpEF [24], mirroring our trial emulation findings and suggesting tirzepatide's GIP component enhances natriuresis and myocardial energetics beyond GLP‐1 alone [25, 26]. These insights question weight loss as the sole mechanism, proposing peak receptor dynamics and lipid/endothelial effects as key differentiators.

### 4.4 Stroke and Atherosclerotic Outcomes

Tirzepatide demonstrated consistent reductions in stroke risk in this study, with relative reductions of 8.2% at 1 year (HR: 0.918) and 9.6% at 3 years (HR: 0.904). These findings align with enhanced cerebrovascular protection and mirror the directional trend toward fewer strokes observed in the SURPASS‐CVOT trial, albeit without reaching statistical significance (HR: 0.91; 95% CI: 0.76–1.09) [12]. Such effects support hypotheses of superior endothelial function and antiplatelet activity conferred by dual GLP‐1/GIP receptor agonism [27]. A large pooled analysis of over 60,000 patients treated with GLP‐1 receptor agonists confirmed significant reductions in major atherosclerotic events, including stroke (HR: 0.83; 95% CI: 0.76–0.92; *p* = 0.0002), cardiovascular death (HR: 0.87; 95% CI: 0.80–0.94; *p* = 0.0010), and fatal or non‐fatal myocardial infarction (HR: 0.90; 95% CI: 0.83–0.98; *p* = 0.020) [28]. These benefits may be mediated, in part, by superior lipid optimization and attenuation of inflammation within the microvascular milieu characteristic of HFpEF.

Myocardial infarction rates remained comparable between groups (1‐year HR: 0.995; 3‐year HR: 0.976), consistent with the predominance of microvascular rather than macrovascular disease in HFpEF [4, 5, 27]. Nonetheless, delayed benefits may emerge with extended follow‐up, reflecting the gradual progression of coronary atherosclerosis [27]. The observed reductions in the composite major adverse cardiovascular events (MACE) endpoint (11.5% relative reduction at 1 year; 13.7% at 3 years) exceeded those reported in the SURPASS‐CVOT trial (HR: 0.92; 95.3% CI: 0.83–1.01; *p* = 0.003) [12].

### 4.5 Renal and Safety Outcomes

Tirzepatide exhibited a robust renoprotective profile, with relative reductions in major adverse kidney events of 15.5% at 1 year, escalating to 20.9% at 3 years, underscoring its potential along the cardiorenal axis in HFpEF. This aligns with findings from the SURPASS‐CVOT trial demonstrating superior preservation of estimated glomerular filtration rate (eGFR) versus dulaglutide in patients with high‐ or very‐high‐risk chronic kidney disease (eGFR decline: 5.72 mL/min/1.73 m^2^ in the tirzepatide group *vs.* 8.90 mL/min/1.73 m^2^ in the dulaglutide group; difference, 3.17 mL/min/1.73 m^2^; 95% CI: 2.09–4.26) [12], potentially mediated by GIP receptor agonism conferring additional glomerular benefits [15, 29].

A modest, marginally‐significant 3.6% relative increase in atrial fibrillation was observed, possibly attributable to hemodynamic or electrolyte shifts induced by rapid weight loss [30], warranting vigilant monitoring without detracting from overall benefits. The risk of acute pancreatitis was lower with tirzepatide compared with other GLP‐1 receptor agonists, alleviating class‐level concerns [31], while hypoglycemia remained neutral, consistent with its glucose‐dependent mechanism of action [13]. Beyond efficacy, tirzepatide exhibited a favorable safety profile relative to dulaglutide, with reduced pancreatitis (22.5% at 1 year; 18.5% at 3 years) despite higher gastrointestinal tolerability issues in trials [12]. This may reflect real‐world dose titration mitigating risks. Hypoglycemia equivalence (HR ~ 0.95) aligns with both agents’ safety in T2DM [9, 10], crucial for elderly HFpEF patients.

### 4.6 Strengths of the Study

This study possesses several key strengths that bolster its reliability and applicability. With a post‐matching sample size of 48 610 patients, it stands as one of the largest comparative real‐world analyses of incretin therapies in HFpEF. Propensity score matching achieved excellent covariate balance, with standardized mean differences below 0.10 for the majority of over 50 demographic, clinical, laboratory, and treatment variables, effectively minimizing measured confounding and enhancing causal inference in an observational setting. The active‐comparator, new‐user design mitigated indication and immortal time biases common in pharmaco‐epidemiologic studies, while strict exclusion of prior comparator exposure ensured clean cohorts free from carryover effects. The inclusion of extended 3‐year follow‐up in a subpopulation with continuous EHR activity allowed for robust evaluation of treatment durability, a feature seldom available in GLP‐1RA observational research and critical for chronic conditions like HFpEF. Comprehensive outcome ascertainment, including all‐cause mortality via linkage to national death registries, all‐cause hospitalization through detailed procedural codes, and composite endpoints like MACE and MAKE, provided clinically meaningful metrics validated in prior CVOTs [12]. Finally, the real‐time federated data from >250 million patients across 69 U.S. HCOs offered high generalizability within diverse clinical practices, complementing RCT evidence by capturing broader patient heterogeneity [32].

### 4.7 Limitations of the Study

Despite these strengths, several limitations must be acknowledged to contextualize the findings. The retrospective observational design inherently cannot fully eliminate residual or unmeasured confounding from variables not captured in EHRs, such as socioeconomic status, NYHA functional class, frailty indices, medication adherence, specific dose titration patterns, or concurrent lifestyle interventions (e.g., diet or exercise programs), even with rigorous propensity score adjustment incorporating >50 covariates. Diagnoses were ascertained using ICD‐10‐CM codes without central adjudication or manual chart review, which may have led to some degree of misclassification. In particular, the definition of heart failure with preserved ejection fraction (HFpEF) relied primarily on the ICD‐10‐CM code I50.3. Although we performed a sensitivity analysis restricted to patients with a recorded diagnosis of diastolic (congestive) heart failure (I50.3) and LVEF ≥ 50% on the most recent measurement, we did not have access to detailed echocardiographic parameters of diastolic function (such as E/eʹ ratio, left atrial volume, or natriuretic peptide levels in all patients). Consequently, some phenotypic heterogeneity may still exist within the HFpEF population. Data limitations precluded analysis of cause‐specific mortality (e.g., cardiovascular vs non‐cardiovascular), granular characterization of heart failure events (inpatient admissions vs outpatient worsening), patient‐reported outcomes like Kansas City Cardiomyopathy Questionnaire scores or 6 min walk distance, and serial changes in key mechanistic variables such as body weight, BMI, NT‐proBNP, or inflammatory markers, hindering deeper interpretation of tirzepatide's benefits. Information on medication persistence, discontinuation rates, achieved doses, and concomitant therapies beyond baseline was unavailable, which may differentially affect long‐term effectiveness estimates, particularly given tirzepatide's titration requirements and potential for gastrointestinal intolerance. Lastly, the study's predominant reliance on U.S. healthcare organizations may limit generalizability to international settings with varying demographics, healthcare systems, prescribing patterns, or access to incretin therapies [33].

#### 4.7.1 Implications and Future Directions

These findings position tirzepatide as a potentially preferred incretin agent in HFpEF management, challenging assumptions of class uniformity and advocating for personalized selection based on dual agonism's pharmacokinetic advantages [12]. The observed superiority, despite dulaglutide's established profile [10], suggests GIP co‐activation enhances cardiorenal and metabolic effects critical for HFpEF's pathophysiology. Clinically, this supports prioritizing tirzepatide in high‐risk patients with obesity, diabetes, or cardiorenal overlap, potentially as adjunct to SGLT2 inhibitors, while monitoring for atrial fibrillation in arrhythmia‐prone individuals [3, 9]. Mechanistic investigations are warranted to elucidate GIP's contributions to natriuresis, endothelial function, and myocardial energetics, perhaps through biomarker substudies or imaging trials [16]. Randomized head‐to‐head trials powered for hard endpoints such as tirzepatide versus dulaglutide in obese/non‐obese HFpEF are urgently needed to confirm these observational signals. Until then, integrating this real‐world evidence with ongoing registries and network meta‐analyses could refine guidelines for GLP‐1RA selection in this heterogeneous syndrome [23].

## 5 Conclusion

In this propensity score–matched real‐world study of 48 610 patients with HFpEF, tirzepatide showed superior outcomes versus dulaglutide at 1‐ and 3‐year follow‐up, with marked reductions in all‐cause mortality, hospitalization, stroke, MACE, MAKE, and acute pancreatitis; benefits accruing over time. These findings extend SURPASS‐CVOT's noninferiority signal, emphasizing dual GLP‐1/GIP agonism's advantages in obesity‐driven HFpEF, independent of diabetes. These hypothesis‐generating data challenge GLP‐1RA class uniformity and call for randomized head‐to‐head trials to guide personalized therapy.

## Author Contributions

I.K. conceptualised and designed the study, performed all data extraction and statistical analyses using the TriNetX platform, created all tables and figures, wrote the initial draft, and critically revised the manuscript for important intellectual content. M.I.H. contributed to study conceptualisation, interpretation of results, and critical revision of the manuscript. F.W. assisted with data verification, contributed to the interpretation of cardiovascular outcomes, and reviewed the manuscript. M.M.A. participated in literature review, drafting of the methods section, and manuscript review. A.A.K. contributed to literature review, drafting of the introduction section, and critical revision of the manuscript. S.A.P. contributed to literature review and manuscript review. M.A.S. participated in literature review and critical revision. M.T.R., and P.S. provided senior methodological oversight on propensity‐score matching and real‐world data analysis, contributed to interpretation of results, and critically revised the manuscript for important intellectual content. All authors reviewed and approved the final version of the manuscript and agreed to be accountable for all aspects of the work. I.K. is the guarantor and accepts full responsibility for the overall content.

## Funding

The authors have nothing to report.

## Ethics Statement

This study utilized fully de‐identified, aggregated electronic health records from the TriNetX US Collaborative Network. All analyses were performed on anonymized data in compliance with the Health Insurance Portability and Accountability Act (HIPAA) and General Data Protection Regulation (GDPR), obviating the need for institutional review board approval or individual patient consent.

## Consent

Not applicable. The manuscript contains no individual person's data, details, images, or videos.

## Conflicts of Interest

The authors declare no conflicts of interest.

## Supporting information

Supporting File

## Data Availability

All data generated and analysed during this study were derived from the TriNetX US Collaborative Network under a limited‐use agreement that prohibits redistribution of individual‐level or raw data. Aggregate‐level results, including cohort characteristics, outcome counts, event free survival probabilities, and statistical summaries, are available within the published article and its supplementary materials.
