# Supplementary material for: Superior Cardiorenal Protection With Tirzepatide Over Dulaglutide in Heart Failure With Preserved Ejection Fraction: A Large‐Scale Propensity Score–Matched Real‐World Analysis
Source: Clin Cardiol. 2026 Aug 10;49(8):e70441. doi: 10.1002/clc.70441 (PMC13455117; doi:10.1002/clc.70441)
Supplement: Supplementary file 1 [file CLC-49-e70441-s001.docx]

**Table S1: Approximate Characteristics of Unmatched Patients**

|  | Characteristic | Tirzepatide Unmatched Mean | Dulaglutide Unmatched Mean | Difference (Tirz – Dula) | Interpretation (Unmatched Tirzepatide vs Dulaglutide) |
| --- | --- | --- | --- | --- | --- |
| 1 | Age (years) | 60.92 | 71.51 | –10.59 | Younger in unmatched Tirzepatide group |
| 2 | BMI (kg/m²) | 41.47 | 35.52 | +5.95 | Higher BMI in unmatched Tirzepatide group |
| 3 | Body weight (lbs) | 261.60 | 223.27 | +38.33 | Heavier in unmatched Tirzepatide group |
| 4 | Creatinine (mEq/L) | 1.16 | 1.43 | –0.27 | Lower creatinine in unmatched Tirzepatide |
| 5 | Sodium (mEq/L) | 139.44 | 138.21 | +1.23 | Slightly higher sodium in unmatched Tirzepatide |
| 6 | Heart rate (bpm) | 77.10 | 78.62 | –1.52 | Slightly lower heart rate in unmatched Tirzepatide |
| 7 | Systolic BP (mmHg) | 126.85 | 132.64 | –5.79 | Lower systolic BP in unmatched Tirzepatide |
| 8 | Hemoglobin (g/dL) | 13.49 | 12.49 | +1.00 | Higher hemoglobin in unmatched Tirzepatide |
| 9 | HDL cholesterol (mg/dL) | 45.95 | 40.50 | +5.45 | Higher HDL in unmatched Tirzepatide |
| 10 | NT-proBNP (pg/mL) | 1190.40 | 2440.01 | –1249.61 | Much lower NT-proBNP in unmatched Tirzepatide |
| 11 | LVEF (%) | 56.28 | 53.93 | +2.35 | Slightly higher LVEF in unmatched Tirzepatide |

**Table S2: Sensitivity analysis showing the Kaplan-Meier survival analyses for all clinical outcomes comparing Tirzepatide versus Dulaglutide in propensity score-matched cohorts (n = 4,141 per group) at 1-year follow-up. The analysis was restricted to patients with a diagnosis of diastolic (congestive) heart failure (ICD-10-CM I50.3) and preserved left ventricular ejection fraction (LVEF ≥50%, based on the most recent measurement).**

| No. | Outcome | Tirzepatide Cohort Events (%) | Dulaglutide Cohort Events (%) | Tirzepatide Cohort KM Survival Probability at 1-Year of Follow-up | Dulaglutide Cohort KM Survival Probability at 1-Year of Follow-up | Hazard Ratio (Tirzepatide vs Dulaglutide) | HR 95% CI | Log-Rank p-value | Proportionality Test p-value |
| --- | --- | --- | --- | --- | --- | --- | --- | --- | --- |
| 1 | All-cause Mortality (ACM) | 90 (2.17%) | 232 (5.60%) | 96.96% | 93.48% | 0.465 | 0.364–0.593 | <0.001 | 0.629 |
| 2 | Acute Heart Failure | 673 (16.25%) | 1,072 (25.89%) | 79.40% | 71.55% | 0.661 | 0.600–0.728 | <0.001 | 0.004 |
| 3 | All-cause Hospitalization | 1,164 (28.11%) | 1,918 (46.32%) | 64.30% | 50.49% | 0.585 | 0.544–0.629 | <0.001 | 0.000 |
| 4 | Myocardial Infarction (MI) | 246 (5.94%) | 404 (9.76%) | 92.12% | 88.97% | 0.692 | 0.591–0.812 | <0.001 | 0.322 |
| 5 | Stroke | 543 (13.11%) | 671 (16.20%) | 82.58% | 81.57% | 0.927 | 0.828–1.039 | 0.192 | 0.367 |
| 6 | Atrial Fibrillation (Afib) | 1,224 (29.56%) | 1,406 (33.95%) | 65.14% | 63.45% | 0.917 | 0.849–0.990 | 0.027 | 0.052 |
| 7 | MACE | 489 (11.81%) | 692 (16.71%) | 84.47% | 81.29% | 0.794 | 0.707–0.892 | <0.001 | 0.062 |
| 8 | MAKE | 199 (4.81%) | 388 (9.37%) | 94.19% | 89.73% | 0.557 | 0.469–0.660 | <0.001 | 0.750 |

**Figure S1: Empirical CDF plot showing density distributions of propensity scores before and after 1:1 greedy nearest-neighbor propensity score matching with a caliper of 0.1 in patients with heart failure with preserved ejection fraction (HFpEF).**
